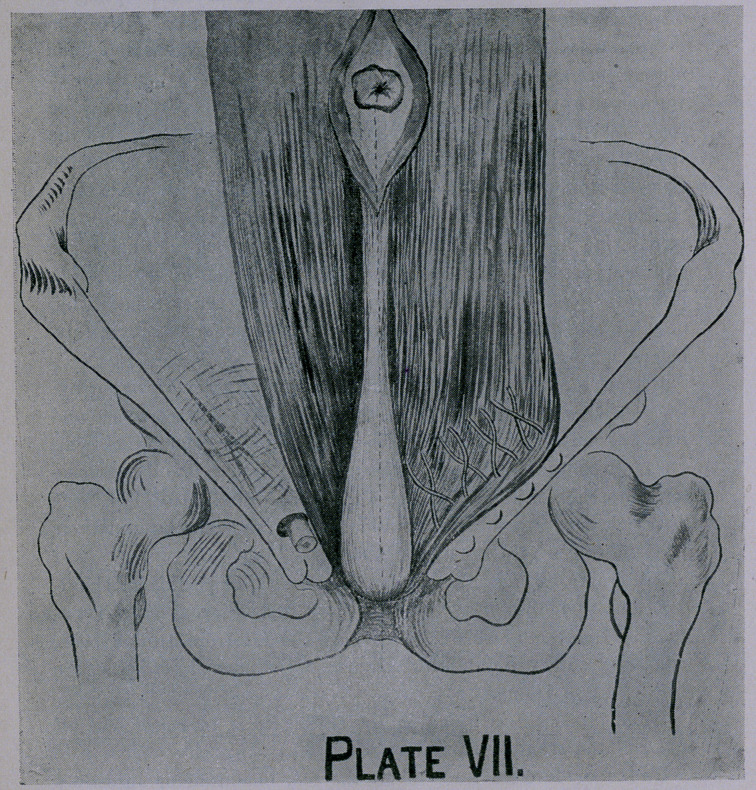# The Anatomic Operation for Radical Cure of Inguinal Hernia under Local Anesthesia*Read at meeting of Houston County Medical Society, September, 1908.

**Published:** 1908-09

**Authors:** O. L. Norsworthy

**Affiliations:** Houston, Texas


### Original Articles.

For Texas Medical Journal.

### The Anatomic Operation for Radical Cure of Inguinal Hernia Under Local Anesthesia.*

BY O. L. NORSWORTHY, HOUSTON, TEXAS.

In May, 1900, at the meeting of the American Medical Association in Columbus, Ohio, Dr. Alexander Hugh Ferguson, of Chicago, presented the anatomic operation for the radical cure of

*Read at meeting of Houston County Medical Society, September, 1908.

inguinal hernia (without reference to the anesthetic used), which was the first to go to print describing it; since which time the operation has been known as the typic, anatomic or Ferguson's operation.

While the anatomical principles on which the typic operation is founded are as unassailable and as fixed as the anatomy of the part itself, yet the technique may vary according to the condition encountered in each individual case. The aim of the opera-

PLATE I.

tion is to imitate nature by fixing the various structures in their proper relationship one to the other. Even when the anatomy is defective (congenital or acquired) the surgeon who possesses a practical knowledge of the normal parts in the inguinal region, and if an experienced operator, should have but little difficulty in differentiating one structure from the other, and execute a rational procedure which takes into consideration the function of each anatomic structure in this area.

According to Dr. Ferguson* the congenitally deficient origin of the internal oblique and transversalis muscles at Poupart's ligament is one of the most frequent and important causes of oblique inquinal hernia. In oblique hernia the internal ring receives no substantial protection from the internal oblique muscle on account of not being attached to the internal aspect of Poupart's ligament sufficiently low down. As the muscle passes downward and inward

PLATE II.

from its deficient origin it passes above the center of the internal ring. It is the congenital deficient origin of this muscle which causes the non-closure of the internal ring. (Plate I.)

Dr. Ferguson's assistant made fifty dissections on cadavers, the result of which supported the Ferguson claim that the deficient

---

*Modern Operations for Hernia, A. H. Ferguson, page 283.

origin of the internal oblique and transversalis muscles at Poupart's ligament was always present in oblique inguinal hernia.

The two main structures that stand on guard to protect the internal ring are the internal oblique muscle and the aponeurosis of the external oblique. The internal oblique is the active agent ready at any time to contract instantly the moment intra-abdominal pressure is suddenly increased.

PLATE III.

The key to the radical cure of inguinal hernia is ablate the sac and protruding omentum, if any; remove all fat; reduce the slack in the tissues surrounding the upper portion of the cord; suture the transversalis and internal oblique muscles to the inner ledge of Poupart's ligament as low down as possible without undue tension, a little more than two-thirds the way down in the male and still more in the female, which is about their normal attachments; stitch securely the flaps of the aponeurosis of the external oblique muscle together; coapt well the superficial fascia. Do not interfere with the normal course of the cord.

It is solely upon anatomical grounds that the operation for

PLATE IV.

oblique inguinal hernia lends itself peculiarly to local anesthesia.

The nerves supplying sensation to the entire area involved in an operation for oblique inguinal hernia are the ileo-inguinal, ileo-hypogastric and genito-crural. Early operators endeavored to cocainize all three nerves, but experience has taught that it is unnecessary. Cocainization of the ileo-inguinal nerve alone with infiltration into certain well-known sensitive areas suffices for a painless dissection.

*Operation.*—After careful aseptic preparations thoroughly infiltrate the skin and subcutaneous tissues with a 1-500 warm cocaine solution, beginning at a point over Poupart's ligament about one inch below the anterior superior spinous process of the

PLATE V.

ilium and continuing inward and downward in a curved (or straight) line, terminating it over the pubic bone.

Your incision can now be made down to and through the external oblique aponeurosis without pain to the patient. With this infiltration you cocainize the tissues supplied by the anterior cutaneous branch of the ileo-hypogastric nerve in this immediate area, and if you do not carry your dissection quite to or below the external ring the patient scarcely knows that you have cut him.

Be especially careful to use sharp knives, clean cutting scissors, and make quick, clean cuts; dull knives, chewing scissors, clamping large areas of tissue and dragging on soft parts are calculated to produce some pain and favor suppuration in the wound.

Use mosquito-bill forceps, pick up each vessel before cutting it,

PLATE VI.

and ligate to prevent blood staining the tissue and blurring the anatomy. By using the curved incision it is not necessary to cut the superficial circumflex iliac nor the superficial pubic vessels.

Turn your flap of skin, subjacent fat and superficial fascia outward and downward over the thigh; this brings into view the aponeurosis of the external oblique muscle, the external abdominal ring with its pillars and inter-columnar fibers, external surface of Poupart's ligament and the sac if protruding through the external ring. (Plate II.)

Now cut through the inter-columnar fiber, split the longitudinal fibers of the aponeurosis of the external oblique muscle directly over the inguinal canal beyond the internal ring.

Retract the upper flap of the aponeurosis of external oblique muscle and cocoainize the ileo-inguinal nerve before attempting to dissect farther. (Plate II.)

This nerve can be found after it pierces the internal oblique muscle just anterior to the anterior superior spine of the ileum in the upper angle of the incision. In most cases it can readily be seen traveling down the internal oblique muscle directly over the inguinal canal and splitting up into filaments as it approaches the external ring. (Plate II.)

The tissues immediately surrounding the cord, sac, and external ring are supplied by filament from the ileo-inguinal nerve and are rich in small blood vessels; it is, therefore, essential to infiltrate the tissues immediately surrounding the external ring, neck of sac and fibers of the internal oblique and transversalis before farther dissection.

Retract the two flaps of the external oblique aponeurosis, bringing into view the contents of the inguinal canal, the whole sac and its adhesions, the spermatic cord, the internal abdominal ring (usually enlarged), an accumulation of subserous fat, the cremasteric muscle, conjoined tendon, the internal surface of Poupart's ligament, the internal oblique and transversalis muscles and their deficient origin at Poupart's ligament. (Plate II.)

Preferably open the sac at its neck near the peritoneal shoulder, and then dissect it from the cord and internal ring downwards. Ligate and ablate sac, remove the fat from around neck, canal and cord without interfering with the normal course of the cord. (Plate III.)

When omentum is found in the sac ligate it *en masse,* cut off and roll the stump up within itself.

Undue or forcible traction on the sac, omentum or intestines will likely cause some little dull pain and slight nausea.

I have had these symptoms to arise and recently in only one case did the patient vomit.

Now inspect carefully here, and if the structures are well defined and not too much weakened by pressure atrophy an anatomic operation can be performed. (Plate III.)

It is here we notice so plainly the deficient origin of the internal oblique muscle at Poupart's ligament as a prominent cause of hernia. (Plate III.)

The redundancy of the cremasteric muscle is taken up by a separate stitch (Plate V), or is sutured with the transversalis and internal oblique muscles to Poupart's ligament with the same stitch, which makes a new and better protected ring (Plate VI). The

PLATE VII.

external oblique aponeurosis is now stitched either by overlapping or not.

Coapt well the cellular superficial fascia and skin and apply a good broad spica bandage.

The cord must not be diverted from its normal course.

It is admitted that over 6 per cent of the recurrences in Bassini's operation occur at the upper angle of the wound, which is due to transplanting the cord at that point.

Be careful not to go too deep with your needle for fear of injuring the deep epigastric vessels or large veins. (Plate V.)

Do not grasp the same longitudinal fibers of Poupart's ligament with each needle bite for fear of splitting the ligament.

If the conjoined tendon be deficient or a direct hernia co-exist the sheath of the rectus muscle is opened freely down to the pubic bone and the muscle is brought across the weak point and stitched to Poupart's ligament; then follow the same procedure for completing the operation as you do in oblique hernia. (Plate VII.)

*Advantages of Local Anesthesia in Hernia Operations.*—1. It avoids the danger of general anesthetic, also avoids all symptoms which sometimes follow any general anesthetic, i. e., nausea, vomiting, distention and intra-abdominal pressure.

2. You are less likely to cut the nerves supplying the muscles to be used for correcting the hernia.

3. More patients will submit to a cure on account of no general anesthetic.

4. The patient being wide awake can cough or strain the sac down, sometimes aiding the operator during the operation.

5. We are less likely to have suppuration follow on account of clean-cut dissection and less blunt dissection and handling of the tissues.

*Limitations.*—I have found it very hard and uncertain to cocainize fat tissue and in more than one instance have I been forced to cause a fat patient pain or give some general anesthetic to complete the operation. I have failed to complete the operation without some general anesthetic toward the end of a prolonged dissection for adhesions around the cord and sac; especially have I found this true in cases coming from the hands of men who practice the injection of irritants as a treatment. In these cases the anatomy is so disfigured, the adhesions so numerous and dense and the dissection necessarily so prolonged it is almost impossible to complete a painless operation under local anesthesia alone.

*Suture Material.*—All vessels should be tied with small (No. 0) plain sterile catgut.

All muscle stitching should be done with chromicized catgut No. 0 or No. 1.

Ligate the sac with No. 1 or 2 chromicized catgut.

EFFECTS OF WINE UPON TYPHOID BACILLI.—Sabrazé and Mercandier, in a communication to the *Annales de l'Institut Pasteur* (April, 1907), report that by experimentation they had found that claret wine kills the bacillus of Eberth in two hours, in its pure state, and in four hours when diluted one-half with water. White wine kills the same micro-organisms in twenty minutes. Champagne destroys them in ten minutes. Used as a disinfecting agent for drinking water, which, owing to failure in boiling or imperfect filtration, is suspected of containing pathogenic germs, the mixture of wine and water should be made six hours in advance of the meal in case of white wine, or twelve hours in the case of red wine, instead of diluting the wine at the table. The old established practice of diluting the wine in cask believed to be followed by some "wine dealers" is therefore not without a certain justification.—*Journal de médecine de Bordeaux.*

---

ENTERITIS.—

℞ Liq. potassii arsenitis........................fl. ℥j

Sig. Two to three drops in water after meals.

Indications.—Used in chronic catarrhal and membranous enteritis.

Also:

℞ Sodii phosphatis . ........................℥viij

Sig. Two teaspoonfuls in glass of hot water before meals.

Indications.—Used in membranous enteritis with constipation.

Also:

℞ Argenti nitratis . ..........................ʒij

Aq. dest...................................fl. ℥xvj

M. Sig. One ounce to pint of distilled water injected into colon after thoroughly irrigating with tepid water. If very painful, follow by injection of weak salt solution.

Indications.—Used in chronic catarrhal and membranous enteritis.—*Ex.*

---

DR. JNO. A. ARMSTRONG, city physician of San Marcos, Texas, died at his home in that city August 20, 1908.

---

MIKE—"And poor Patrick is dyin' of the insomny, they say."

Dennis—"And how did he get it?"

Mike—"Faith, he got it lying awake o' nights listening to himself talk in his schleep."

## Figures and Tables

**Plate I. f1:**
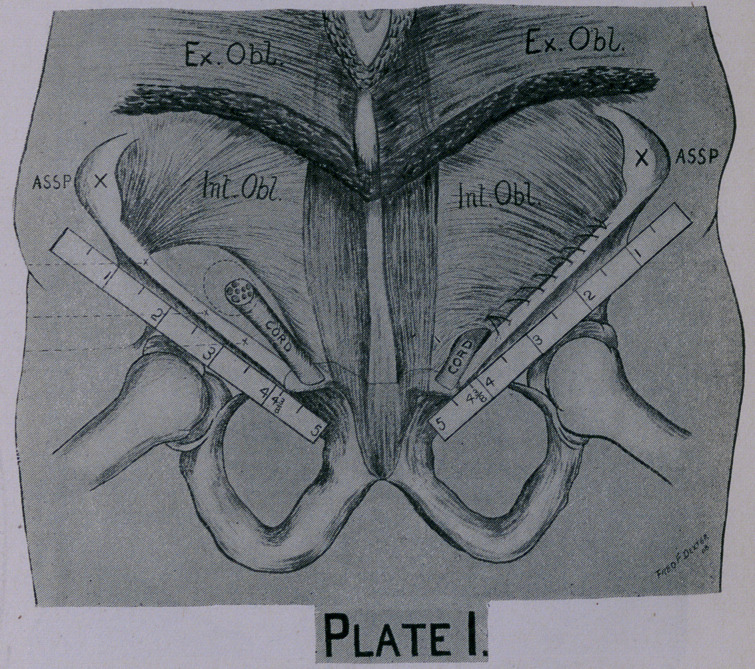


**Plate II. f2:**
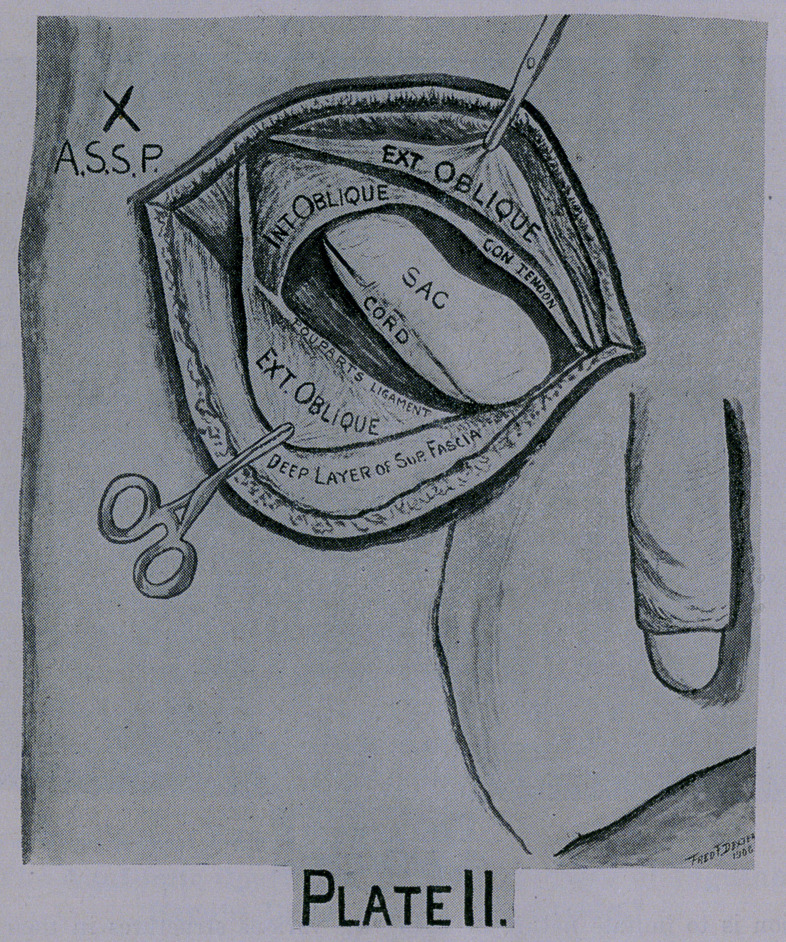


**Plate III. f3:**
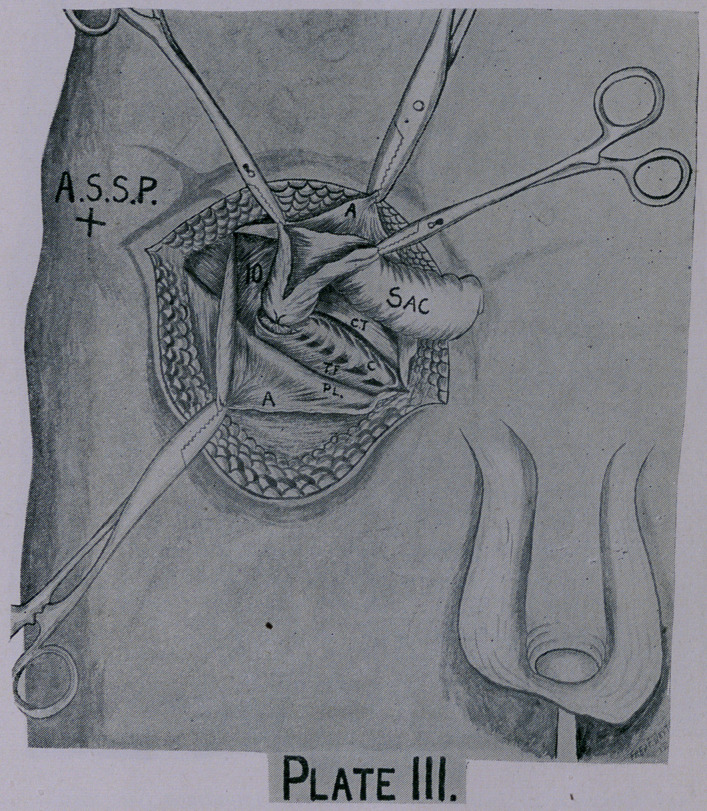


**Plate IV. f4:**
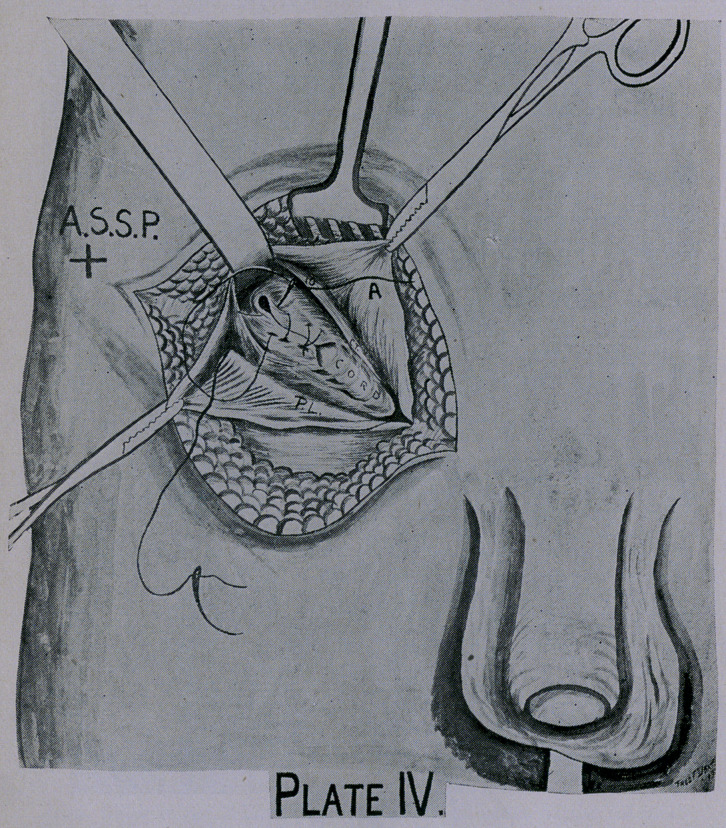


**Plate V. f5:**
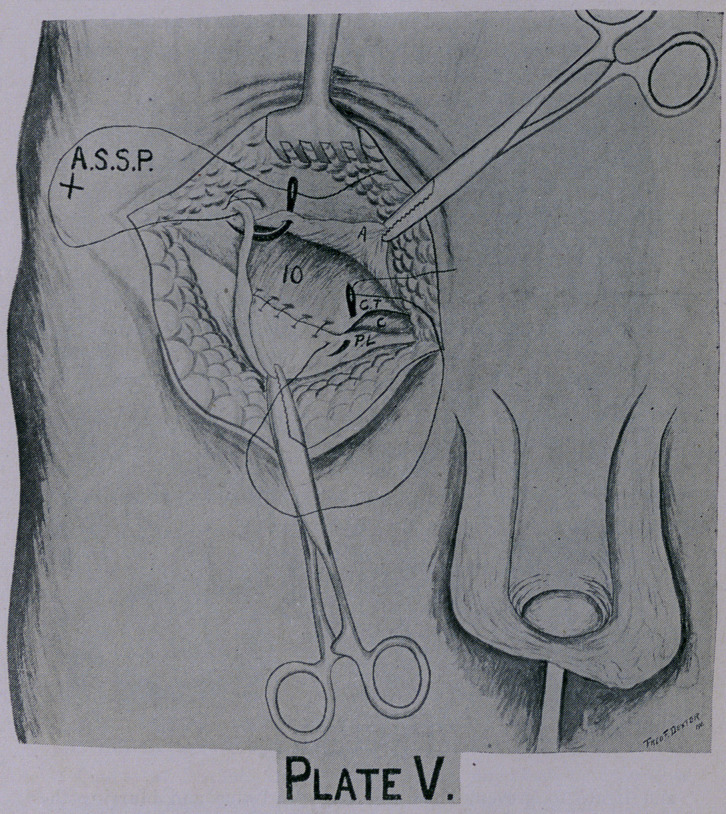


**Plate VI. f6:**
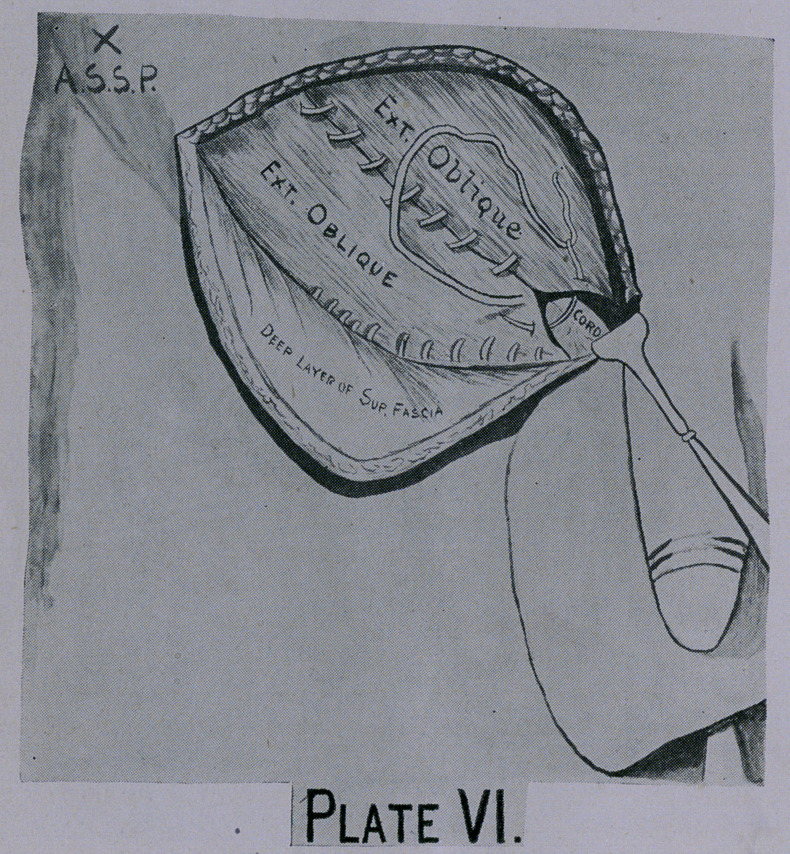


**Plate VII. f7:**